# Prevalence of hepatitis B and C virus infections among patients with chronic hepatitis at Bereka Medical Center, Southeast Ethiopia: a retrospective study

**DOI:** 10.1186/1756-0500-7-272

**Published:** 2014-04-29

**Authors:** Solomon Taye, Awel Abdulkerim, Mohammed Hussen

**Affiliations:** 1 Microbiology, Immunology and Parasitology Department, College of Medicine and Health Sciences, Madawalabu University, P.O. Box 302, Bale Robe, Ethiopia; 2 Department of Internal Medicine, College of Medicine and Health Sciences, Madawalabu University, P.O. Box 302, Bale Robe, Ethiopia; 3 Bereka Medical Center, Bale Robe, Ethiopia

**Keywords:** Hepatitis B virus, Hepatitis C cirus, Chronic hepatitis, Prevalence

## Abstract

**Background:**

Viral hepatitis exists throughout the world and is a major global public health problem affecting millions of people. Hepatitis B (HBV) and hepatitis C (HCV) virus are the commonest causes of inflammation of the liver leading to chronic liver disease, cirrhosis, hepatocellular carcinoma and even fulminant hepatitis. The objective of this retrospective study was to determine the prevalence of infections with HBV and HCV among patients with chronic hepatitis who visited Bereka Medical Center, southeast Ethiopia.

**Methods:**

Institution based retrospective study design was employed. HBV and HCV test records of all patients with chronic hepatitis who visited Bereka Medical Center from Nov. 2012 to Nov. 2013. A total of 578 (358 for HBV and 220 for HCV) patients with chronic hepatitis screened and the results of all patients were included. All sera were screened using commercially available rapid test kits. Test results were shown in percent and prevalence rates.

**Results:**

The overall prevalence of HBV and HCV among chronic hepatitis patients were 22.3% and 3.6% respectively. Prevalence of HBV and HCV among males from the total HBV and HCV screened was 52/358 (14.5%) and 6/220 (2.7%) respectively. 64/80 (80%) of HBV sero-positives were found in between 16 to 45 years of age. Of those 64 HBV sero-positive patients, 42/64 (65.6%) were found 16–30 age interval. Furthermore, of those HCV infected, 6/8 (75%) of them fall under 16–30 years of age. 6/8 (75%) of HCV infected patients were males.

**Conclusion:**

The present study has shown that HBV was highly prevalent among patients who visited the clinic. Males were more susceptible than female patients. 3.6% HCV prevalence was also high. Mass immunization of HBV is recommended to halt HBV infection.

## Background

Viral hepatitis exists throughout the world and is a major global public health problem. It is an inflammation of the liver leading to chronic liver disease, cirrhosis, hepatocellular carcinoma and even fulminant hepatitis. The most common causes are hepatitis B virus (HBV) and hepatitis C virus (HCV) [1]. HCV is an enveloped single-stranded positive sense RNA virus belonging to the genus Hepacivirus within the family *Flaviviridiae*[2] and HBV is a partially double-stranded circular DNA belonging to the family *Hepadnaviridae* in the genus *Orthohepadnavirus*. HBV causes acute hepatitis of varying severity and persists in 95% of children and 10% of adult patients [3]. In Ethiopia, 12% of hospital admissions and 31% of mortalities in medical ward are due to liver disease [4].

Both virus share common mechanism of transmission (parental, sexual and mother to child) and HBV is 50 to 100 times more infectious than HIV [4,5]. It is estimated that more than 2 billion people in the world have been infected with HBV. Of these, more than 360 million are chronically infected and they are at risk of serious illness and death from cirrhosis and hepatocellular carcinoma [6]. The burden of HBV infection is highest in the developing world particularly in Asia and sub-Saharan Africa. WHO estimated that the prevalence of HBV infection in Africa is on average more than 10%. However, a study conducted in Addis Ababa showed that the mean prevalence of HBsAg was 6.1% [7,8].

WHO estimated also that approximately 170 million people are infected with HCV and about 130 million are carriers and three to four million persons are newly infected each year and more than 350,000 people estimated to die from HCV-related liver diseases each year worldwide. HCV infection in the world varies from 0.3 to 13% or more with the highest prevalence recorded in Central Africa and South-Eastern Asia. However, a study in Addis Ababa showed that 0.9% prevalence among population and 1.3% among adults over 15 years of age [8,9].

In the present study area, the information on the prevalence of those hepatovirus is not known. Thus, the aim of this study is to determine the prevalence of HBV and HCV among patients with chronic hepatitis who visited Bereka Medical Center, southeast, Ethiopia.

## Methods

The study was conducted at Bereka Medical Center in Bale Robe town, bale zone, Oromia regional state, southeast, Ethiopia. Institution based retrospective study design was used and the screening data’s of HBV and HCV of all patients with chronic hepatitis who visited the clinic from Nov 2012 to Nov 2013 were considered for the study. All patients with chronic hepatitis were screened for possible hepatoviral infection. During the study period, a total of 578 (358 for HBV and 220 for HCV) patients were screened for HBsAg and anti- HCV and the results all patients were included for the present study.

Standard procedures were followed during blood sample collection. All sera were screened for HBsAg and antibodies to HCV using commercially available screening rapid test kits according to the manufacturer's instruction (Qualpro Diagnostics, 88/89, phase IIC, Verma Industrial Estate, Verna, Goa-403 722, India). The test kit has a sensitivity of 100% and specificity of 99.7%. Positive serum samples were assayed in duplicate to increase the quality of the procedure.

Data entry and analysis was done using SPSS version 16 computer software. Data was summarized and presented in a descriptive measure such as a table, figures, mean and percent.

The study was approved by the Research Ethics and Review Committee of Madawalabu University. Records of patients were manipulated only by researchers. HBV and HCV-positive cases were contacted with nurses and doctors for further management.

All patients involved in this report were provided a written informed consent prior to data collection. Furthermore, written informed consent was obtained from the each patient for publication.

## Result

A total of 578 (358 for HBV and 220 for HCV) patients with chronic hepatitis were included for the purpose of this retrospective study. The age of patients’ ranges from twelfth to fifty two years and the mean age of the study population were 34 years. Male to female ratio of the patients were 1.2:1 and 1.3:1 for HBV and HCV respectively.

Blood screening results showed that hepatoviral infections were common among patients having chronic hepatitis who visited the clinic. In the present retrospective study, the overall prevalence of HBV and HCV was 22.3% and 3.6% respectively among patients with chronic hepatitis. Prevalence of HBV among males from the total HBV screened was 52/358 (14.5%) and from the total screened males was 52/198 (26.3%). 64/80 (80%) of HBV positives were found in between 16 to 45 years. Of those 64 HBV positive patients, 42/64 (65.6%) were found 16–30 age group and further 40/64 (62.5%) of them are males. Four patients infected with HBV were children under the age of 15 years and there were no infected children in this age group (Table 1).

**Table 1:** Distribution of chronic hepatitis B and C virus infected patients based on gender and different age intervals at Bereka Medical Center, Southeast, Ethiopia (Nov 2012-Nov 2013)

**Hepatovirus**	**Age group**								**Total positive**	**Total examined**	
	**< 15**		**16-30**		**31-45**		**>46**			**Male**	**Female**
	**Male**	**Female**	**Male**	**Female**	**Male**	**Female**	**Male**	**Female**			
HBV	4	-	24	18	16	6	8	4	**80**	198	160
**Total**		**4**		**42**		**22**		**12**			**358**
HCV	-	-	4	2	2	-	-	-	**8**	124	96
**Total**		**-**		**6**		**2**					**220**

HBV - Hepatitis B virus; HCV – Hepatitis C virus.

Whereas the prevalence of HCV from the total HCV screened and from the total screened males was 6/220 (2.7%) and 6/124 (4.8%) respectively. All of the infected patients were found in the age groups 16–30 and 31–45. Furthermore, of those HCV infected, 6/8 of them fall under 16–30 years of age. 6/8 (75%) of patients were males and none of patients under the age of 15 and > 45 were infected (Table 1). In areas where viral hepatitis is highly prevalent, co-infection of hepatoviral pathogens is common. HBV/HCV co-infection in the present study was 3/62 (4.8%).

## Discussion

Hepatitis B and C virus infections are one of the major diseases of mankind and are a serious global public health problem [1,9]. The present retrospective study aimed at determining the prevalence’s of HBV and HCV infections among patients with chronic hepatitis who visited Bereka Medical Center, from Nov 2012- Nov 2013.

In this study, the overall prevalence of HBV was 80 (22.3%) which was much higher than the previous studies conducted in different parts of Ethiopia; Bulle Hora (0.9%) by Amaha *et al*., Shashemene (5.7%) by Asfaw *et al*. and Gondar (1.0%) by Belay *et al.*[6,8] and other countries; Angola (15.1%) by Fatima *et al.*[7]. In contrast, the present study is very much lower when compared with the study conducted in Kumasi, Ghana (71.0%) by Jean-Pierre Allain *et al*. [10]. These differences in prevalence could be due to the place and living standard of study subjects or due to a reflection of the local endemicity and life style of the study area.

Unavailability of HBsAg mass immunization schemes makes HBV infection a major public health problem globally and especially in sub-Saharan countries [11]. The same is true for Ethiopia and study area which accounts the high prevalence because of unsafe sexual intercourse, lack of specific confirmatory test in blood bank and sharing sharp materials in unaware community.

Hepatitis C virus prevalence (3.6%) in the present study was high when compared with other studies in Ethiopia. The prevalence of HCV is quite different among different communities. For instance, Taye and Lakew study among HIV patients in Addis Ababa showed a prevalence of 6.5% [9]. Another study at Gondar town health institutions northwest Ethiopia indicated 0.0% among medical waste handlers [8] which was very low compared with the present study.

In western countries, HCV is highly prevalent primarily because of injection drug use. Whereas in Ethiopia the habit of injection drug use is very low thus might be responsible for low HCV prevalence of HCV in the community [9].

Furthermore, a study by Taye and Lakew and others clearly indicated HCV infection increases with age increase [9]; however, in this study its prevalence was high in the age group 16–30 years and as age increases the infection rate decreases (Table 1). The reason may be related to the effect of hormone and immunity.

## Conclusion

The present study has shown that HBV was highly prevalent among patients with chronic hepatitis whom visited the clinic during Nov. 2012-Nov. 2013. Furthermore, 3.6% HCV prevalence in the present study was also high when compared with other community based studies in Ethiopia. The absence of confirmatory tests for both HBV and HCV might further compromise the sensitivity and specificity of the results.

## Competing interests

The authors declare that they have no competing interests.

## Authors’ contributions

ST* conceptualized and designed the study. ST* performed the laboratory examinations and data collection. ST* analyzed and interpreted the data, drafted the manuscript and critically reviewed the manuscript. AA assisted in analyzing, drafting and reviewing the manuscript. MH assisted during laboratory examinations and data collection. All the authors’ read and approved the manuscript.
